# *Apc* gene suppresses intracranial aneurysm formation and rupture through inhibiting the NF-κB signaling pathway mediated inflammatory response

**DOI:** 10.1042/BSR20181909

**Published:** 2019-03-26

**Authors:** Xian-Liang Lai, Zhi-Feng Deng, Xin-Gen Zhu, Zhi-Hua Chen

**Affiliations:** Department of Neurosurgery, The Second Affiliated Hospital of Nanchang University, Nanchang 330006, China

**Keywords:** Apc gene, Intracranial aneurysm, Inflammatory response, NF-κB signaling pathway

## Abstract

**Background:** Intracranial aneurysm (IA) is a critical acquired cerebrovascular disease that may cause subarachnoid hemorrhage, and nuclear factor-κB (NF-κB)-mediated inflammation is involved in the pathogenesis of IA. Adenomatous polyposis coli (*Apc*) gene is a tumor suppressor gene associated with both familial and sporadic cancer. Herein, the purpose of our study is to validate effect of Apc gene on IA formation and rupture by regulating the NF-κB signaling pathway mediated inflammatory response. **Methods:** We collected IA specimens (from incarceration of IA) and normal cerebral arteries (from surgery of traumatic brain injury) to examine expression of Apc and the NF-κB signaling pathway related factors (NF-κB p65 and IκBα). ELISA was used to determine levels of monocyte chemoattractant protein-1 (MCP-1), tumor necrosis factor-α (TNF-α), interleukin (IL)-1β (IL-1β), and IL-6. IA model was established in rats, and Apc-siRNA was treated to verify effect of Apc on IA formation and rupture. Next, regulation of Apc on the NF-κB signaling pathway was investigated. **Results:** Reduced expression of Apc and IκBα, and increased expression of NF-κB p65 were found in IA tissues. MCP-1, TNF-α, IL-1β, and IL-6 exhibited higher levels in unruptured and ruptured IA, which suggested facilitated inflammatory responses. In addition, the IA rats injected with Apc-siRNA showed further enhanced activation of NF-κB signaling pathway, and up-regulated levels of MCP-1, TNF-α, IL-1β, IL-6, MMP-2, and MMP-9 as well as extent of p65 phosphorylation in IA. **Conclusion:** Above all, Apc has the potential role to attenuate IA formation and rupture by inhibiting inflammatory response through repressing the activation of the NF-κB signaling pathway.

## Introduction

Intracranial aneurysms (IAs) are acquired lesions with a prevalence of 5–10% of the population, a fraction of which rupture develops into subarachnoid hemorrhage with lethal consequences [1]. Although unruptured IAs have a low risk of rupture, interventions are commonly used due to the unfavorable prognosis of intracranial hemorrhage [2]. A classification of aneurysms has been proposed in a report according to the size, small (<5 mm), medium (5–9.9 mm), large (>10 mm), and giant (>25 mm), and the report also pointed out the annual rupture rate of 0.8, 1.2, 7.1, and 43.1%, respectively [3]. The primary goal of IA treatment is to prevent rupture [4], and endovascular treatment is applied for most cases of IA [5]. As a major cerebrovascular disease, IA formation and development result from endothelial dysfunction and vascular smooth muscle cell (VSMC) phenotypic transformation to pro-inflammatory phenotype [6,7]. Recent studies have reported the potential roles of molecular therapeutic targets and relevant signaling pathways in IAs [8,9].

Adenomatous polyposis coli (*Apc*) gene, cloned in 1991, located at chromosome 5q21-22 and containing 15 exons, has been classified as a tumor suppressor, whose activity is implicated in the regulation of the intracellular level of β-catenin within the Wnt signaling pathway [10,11]. The inhibitory role of Apc in cancers is significantly involved in cellular processes including cell division, adhesion, and migration [12]. The genetic changes of the tumor suppressor *Apc* gene are involved in the brain metastasis, the gross deletion of which promotes the brain metastasis [13]. Apc regulates the activation of the Wnt/β-catenin signaling pathway which plays a pivotal role in the development and maintenance of homeostasis of human body [14]. Furthermore, the activation of the Wnt/β-catenin signaling pathway is closely related to nuclear factor-κB (NF-κB) [15]. NF-κB composes a family of transcription factors and participates in a series of activities, including the regulation of immune responses, maturation of immune cells, development of secondary lymphoid organs, and osteoclastogenesis [16]. The NF-κB pathway is a critical link of inflammatory responses [17]. The expression of NF-κB in the IA tissue is significantly increased as compared with that in the normal intracranial arterial tissues [18]. Inhibition of NF-κB activity has been reported to repress the formation and enlargement of IAs [19]. Above all, we intended to find out the role of *Apc* gene in mediating IA formation and rupture through the NF-κB signaling pathway mediated inflammatory response.

## Materials and methods

### Ethical statement

The present study was approved by the Ethics Committee of the Second Affiliated Hospital of Nanchang University. All participants provided written informed consents. All animal experiments were conducted in accordance with the Guide for the Care and Use of Laboratory Animals published by the National Institutes of Health.

### Study subjects

A total of 108 clinical specimens were obtained from patients who underwent incarceration of IA in the Department of Neurosurgery of the Second Affiliated Hospital of Nanchang University from January 2011 to January 2017. As a prerequisite, the life of patients and surgical security were ensured during the process. There were 58 males and 50 females (aged: 24–67 years) with a mean age of 47 years. And 68 patients had ruptured IA and other 40 cases had unruptured IA. There were 44 cases with diameter >5 mm, 40 cases between 5 and 13 mm, and 24 cases >13 mm. In terms of the locations of IA, 13 cases were in anterior cerebral artery (ACA), 29 cases in anterior communicating artery (AcoA), 15 cases in middle cerebral artery (MCA), 37 cases in posterior communicating artery (PcoA), and 14 cases in posterior inferior cerebellar artery (PICA). All cases were confirmed by digital subtraction angiography (DSA), and resected by microsurgery in the Department of Neurosurgery. The arteries of the same side with the surgical approach during the incarceration of IA were obtained. Control group included normal cerebral arteries resected in surgery from 28 patients (16 males and 12 females) with traumatic brain injury. The tissues were fixed for immunohistochemistry examination. Venous blood samples of every patient were collected within 72 h after admission. ELISA was performed to measure levels of tumor necrosis factor-α (TNF-α), monocyte chemoattractant protein-1 (MCP-1), interleukin (IL)-1β (IL-1β) and IL-6 in serum. The patients were excluded: (i) patients without whole-brain DSA (including four vessel angiography of bilateral internal carotid artery and vertebral artery); (ii) patients with extension or dilatation of artery, traumatic, or infectious aneurysm; (iii) patients who received part or whole endovascular occlusion or craniotomy and clipping of IA in other hospitals before angiography and lacked image data before treatment; (iv) patients without 2D or 3D imaging to determine the aneurysm size.

### Immunohistochemistry

Streptavidin-perosidase (SP) immunohistochemistry kit (HSP0001) was purchased from Shanghai Mjol Biotechnology Co., Ltd. (Shanghai, China). The sections were dewaxed, dehydrated with gradient alcohol, and the antigen retrieval was conducted with microwave. Then 3% H_2_O_2_ was used to block endogenous peroxidase activity for 10 min, and then normal goat serum was used to reduce non-specific background for 15 min. The following primary antibodies were added to the sections: rabbit anti-human NF-κB p65 polyclonal antibody (ab19870, 2.5 µg/ml), rabbit anti-human IκBα polyclonal antibody (ab7217, 1:200) and rabbit anti-human Apc polyclonal antibody (ab133397, 2 µg/ml) and incubated at 4°C overnight. All the above antibodies were purchased from Abcam Inc. (Cambridge, MA, U.S.A.). The sections were treated with horseradish peroxidase (HRP) labeled secondary antibody goat anti-rabbit monoclonal antibody (ab6721, 1:1000, Abcam Inc., Cambridge, MA, U.S.A.) for 15 min, followed by SP treatment for 15 min. Then the color was developed with 3,3′-diaminobenzidine (DAB). Next, the sections were washed under water to terminate the reaction and counterstained with Hematoxylin, dehydrated, cleared, and mounted. PBS was used as negative control (NC) to replace the primary antibody. The Apc positive expression was localized in cytoplasm, and the NF-κB p65 and IκBα positive expression was localized in cytoplasm and some in nucleus. Five high magnification fields of view (×200) with at least 100 positive cells were randomly chosen for counting with double blind method. The method of scoring was shown as follows: (A) the staining intensity was determined by cell color and its depth (without staining = 0 point, light yellow = 1 point, pale brown = 2 points, brown = 3 points); (B) proportion of positive cells (negative cells = 0 point, percentage of positive cells ≤10% = 1 point, percentage of positive cells 11–50% = 2 points, percentage of positive cells between 51 and 75% = 3 points, percentage of positive cells >75% = 4 points). Staining score of each case = A × B. The score <3 points was regarded as negative (−), 3–5 points as positive (+), 5–7 points as strongly positive (++), and >7 points as extremely strongly positive (+++).

### ELISA

The experiments were conducted in strict accordance with the instructions of MCP-1 ELISA kit (ab21396, 100 pg/ml), TNF-α ELISA kit (ab6671, 1:1000), human IL-1β ELISA kit (ab100562, 100 pg/ml), rat IL-1β ELISA kit (ab100705, 100 pg/ml), human IL-6 ELISA kit (ab6672, 1:1000), and rat IL-6 ELISA kit (ab100713, 100 pg/ml). All these kits were purchased from Abcam Inc. (Cambridge, MA, U.S.A.). Blood sample was taken from patients, with 0.109 mol/l sodium citrate as anticoagulant (1:10) and allowed to stand at room temperature for 1 h, followed by centrifugation at 3000 rpm for 10 min. Then the supernatant was obtained, transferred into a 1.5-ml test tube and then stored in a −70°C freezer to avoid repeated freezing and thawing. The known antigen was diluted into the above concentration with carbonates coated buffer (pH 9.6), and added into wells (0.1 ml for each) for overnight incubation at 4°C. The next day, the antigen was washed three times. Then 0.1 ml diluted supernatant was added into the above-coated reaction wells, which were incubated at 37°C for 1 h. Correspondingly, blank, negative, and positive control wells were made, which were added with 0.1 ml of freshly diluted DON-HRP–conjugated secondary antibody (Abcam Inc., Cambridge, MA, U.S.A.) and incubated at 37°C for 35–60 min. After a final wash of ddH_2_O (PER 018-1, Beijing Dingguo Changsheng Biotechnology Co., Ltd., Beijing, China), the reaction wells were added with 0.1 ml temporarily prepared *Mycobacterium tuberculosis* (MTB) substrate (EL0001, InnoReagents, Huzhou, Zhejiang, China) and incubated at 37°C for 10–30 min. At last, 50 μl terminating liquid was added into wells to terminate coloring, and the optical density (OD) values were measured at wavelength of 450 nm within 20 min.

### Establishment of IA model in rat

A total of 50 healthy male or female Sprague–Dawley (SD) rats aged 7 weeks and weighing from 180 to 250 g were provided by Shanghai SLAC Laboratory Animal Co., Ltd. (Shanghai, China). The rats were randomly assigned into normal group (10 rats) and IA group (40 rats). The IA model in rat was established as follows [20]. The rats were intraperitoneally anesthetized with 3% pentobarbital sodium (30 mg/kg, the dose was increased when necessary). The left common carotid artery and bilateral posterior renal arteries were ligated under a surgical microscope. The neck and back of rats were shaved and disinfected and a sterile towel was spread. Longitudinal incisions (1.5–2 cm) were made on dorsal bilateral costal margin in rats. One side of the skin was cut first, then entered into the abdominal cavity through subcutaneous and muscle layers. The thumb of the operator was placed on the rat back, and middle and index fingers on the abdomen to touch the kidney, and extrude the incision on kidney with index or middle fingers. Then omentum and other tissues were pushed back into the abdomen with a sterile cotton swab moistened in sterile normal saline to expose the kidney. The operator gently pressed renal pedicle on medial incision with tweezers or wet cotton swab, thus posterior branch of renal artery and vein automatically separating due to tension. Through the space between posterior branch of renal artery and vein, bipolar coagulation was adopted to block posterior branch of artery, or the posterior branch of artery was ligated by a thread. Then a 0/3 thread was ligated around the posterior branch of renal artery. A clear map-shaped ischemic area appeared in the middle and upper parts of the posterior segment of the kidney. Next, the kidney was placed back into the cavity and the peritoneum and skin were sutured. One week later, the rats were all fed 2% saline water replacing drinking water for 3 months. The rats presenting one of the following symptoms were identified with ruptured aneurysm [21]. (i) Weight loss (weight loss of over 10% for 24 h) and a significant reduction in food and water consumption; (ii) forelimb raising and trunk bending; (iii) walking along one side with normal posture; (iv) leaning to one side at rest; and (v) no spontaneous activity. Rats with these symptoms were killed and the remaining ones were killed 3 months after operation. IA tissues were obtained in the operation, which were perfused with PBS and then perfused with glutin-containing blue dye to manifest cerebral artery. Aneurysm was defined as vascular wall whose diameter was longer than that of aorta with partial outward expansion.

### Transfection and grouping

The 40 rats in the IA group were randomly grouped into blank group (10 model rats), NC group (10 model rats transfected with empty plasmid), Apc-siRNA group (10 model rats transfected with Apc-siRNA), and the remaining 10 model rats for further use. Forty rats in the IA group and ten rats in the normal group were raised in specific pathogen-free (SPF) animal laboratory with humidity of 60–65% and at 22–25°C. Rats in the normal group were treated with stereotactic injection of 100 μl PBS once a day. The rats in the blank group were treated with stereotactic injection of 100 μl mixture of PBS and Lipofectamine™ 2000 (Invitrogen Inc., Carlsbad, CA, U.S.A.) once a day. The volume of Lipofectamine™ 2000 was the same as empty vector group. The rats in the NC group were treated with stereotactic injection of 100 μl mixture of empty vector and Lipofectamine™ 2000 (DNA (μg):g Lipofectamine™ 2000 (μl) = 1:3) once a day. The rats in the Apc-siRNA group were treated with stereotactic injection of 100 μl mixture of Apc-siRNA eukaryotic expression vector and Lipofectamine™ 2000 (DNA (μg):g Lipofectamine™ 2000 (μl) = 1:3) once a day. Blood pressure measured 3 days before surgery was regarded as the basic blood pressure. Caudal arterial blood pressure was measured at the 1st, 4th, and 12th week after the carotid resection. After 3 months, the rats in each group were generally anesthetized and the thoracic cavity was opened according to the aforementioned method. The left ventricle was cannulated to the aorta, and the vena cava was cut to release blood. At the same time, the 30 ml normal saline containing heparin sodium (at 37°C) was perfused through the catheter, and then slowly injected 10 ml of 10% paraformaldehyde/0.1 M phosphate buffer (pH 7.4) into the brain through the catheter. After perfusion fixation, the brain was collected following craniotomy. The circulus arteriosus of the skull base was carefully separated and removed under a surgical microscope (×10 to ×16), and the aneurysmal changes were examined under a microscope (×40). The artery with pathological changes was used for pathological examination.

### Blood pressure measurement

The blood pressure meter on the tails of rats (Kent Industrial Co., Ltd, Chino, CA, U.S.A.) was measured before operation and at the 1st, 4th, and 12th week after operation. The method was shown as follows: rats were deprived of water and food 2 h before measurement, which was started at 8:00 a.m. The room temperature was maintained at approximately 25°C, and the incubator should be preheated to reach a temperature of 37°C. The rats were put into the incubator for 10 min to adapt the circumstance, and then a clamp was placed on the root of rat tails. Only after the instrument was adjusted and rats’ beating stabilized, the blood pressure could be measured. The blood pressure of rats was tested three times to obtain the mean value of blood pressure.

### Extraction and conservation of brain tissue and tissue homogenate

Three months later, the rats in the four groups were generally anesthetized and the thoracic cavity was opened using the abovementioned method. The left ventricle was cannulated to the aorta, and 30 ml normal saline containing heparin sodium (at 37°C) was perfused through the catheter, and then slowly injected 10% paraformaldehyde/0.1 M phosphate buffer (pH 7.4) into the brain through the catheter. Spontaneously, the vena cava or the right atrium was cut to release blood. After perfusion fixation, the whole brain was collected with craniotomy. A 10% homogenate of brain was prepared from the 1 g extracted brain tissues ground in lysate solution containing 10 mmol/l NaCl, 10 mmol/l EDTA, 0.5% NP-40, 0.5% sodium deoxycholate (Shanghai Yiji Industries Co., Ltd, Shanghai, China), and 10 mmol/l Tris (pH 7.4, Nanjing Search Biotech Co., Ltd, Jiangsu, China), followed by centrifugation for 10 min at 2000 rpm. Next, the supernatant was obtained and centrifuged at 14000 rpm for 90 min (4°C). The collected precipitate was further suspended in 40 μl double distilled water (DDW) and stored at −20°C.

### Hematoxylin–Eosin staining

Twenty four hours after the operation, vascular tissues of circles of Willis in rats of each group were fixed in 4% paraformaldehyde overnight, then fixed in 4% formaldehyde for 6 h, and finally embedded in paraffin. Next, the embedded brain tissues were made into 3-μm-thick sections and baked at 60°C overnight. The next day, the sections were dewaxed for 20 min in xylene I (14936-97-1, Shanghai Yiji Industries Co., Ltd, Shanghai, China) and xylene II (523-67-1, Shanghai YuDuo Biological Technology Co., Ltd., Shanghai, China), respectively. And then sections were dehydrated in 100, 100, 95, 80, and 70% ethanol with 5 min for each, and washed with distilled water. Afterward, the sections were stained with Hematoxylin (CAS: 474-07-7, Qingdao Jisskang Biotechnology Co., Ltd, Shandong, China) for 10 min, washed with water to return to blue for 15 min and stained with Eosin (RY0648, Qingdao Jisskang Biotechnology Co., Ltd, Shandong, China) for 30 min. Finally, the sections were washed with DDW to wash off the red color, dehydrated with ethanol, cleared with xylene, and mounted with neutral balsam. The optical microscope was employed to observe the histopathological changes of IA tissues with the images obtained using image analysis system (JD801, Jiangsu JEDA Science-Technology Development Co., Ltd., Nanjing, Jiangsu, China). The morphological changes of IA in Hematoxylin–Eosin (HE) staining sections of each group were observed (×400) with randomly chosen images. The experiment was conducted three times.

### Reverse transcription quantitative PCR

Total RNA was extracted from IA tissues according to the instructions of TRIzol kit (Invitrogen Inc., Carlsbad, CA, U.S.A.) by using one-step method. Then the total RNA was dissolved in ultrapure water treated by diethylpyrocarbonate (DEPC, Sangon Biotech Co., Ltd., Shanghai, China). Then OD values at 260 and 280 nm were determined by a UV-visible spectrophotometer (ND-1000, Thermo Fisher Scientific, Waltham, MA, U.S.A.). The quality of total RNA was identified, and the concentration was measured. The extracted RNA was reverse transcribed according to the instructions of the kit (RR037Q, Takara Biotechnology Ltd., Dalian, Liaoning, China) by using two-step method. The reverse-transcription system (20 μl) was composed of 2 μl of 5× PrimeScript Buffer (for Real Time), 0.5 μl PrimeScript RT Enzyme Mix I, 0.5 μl Oligo dT Primer (50 μM), 0.5 μl Random 6 mers (100 μM), 2 μg Total RNA, and added with RNase-free dH_2_O to a final 20 μl system. The reaction conditions were as follows: 37°C for 15 min, 85°C for 5 s, and forever at 4°C. The cRNA obtained from the reverse transcription was observed in a freezer at −80°C. The TaqMan probe method was applied for reverse transcription quantitative PCR (RT-qPCR). And reaction system was conducted in accordance with the kit instructions (MBI Fermentas International Inc., Vilnius, Lithuania). The primer sequences are shown in Table 1. The reaction conditions were pre-denaturation at 95°C for 30 s, denaturation at 95°C for 10 s, annealing at 60°C for 20 s, and extension at 70°C for 10 s with a total of 40 cycles. The reaction system was composed of 12.5 μl Premix ExTaq or SYBR Green Mix, 1 μl Forward Primer, 1 μl Reverse Primer, 1–4 μl cDNA, and added with ddH_2_O to a final 25 μl system. Real-time fluorescence quantitative PCR (iQ5, Bio-Rad, Hercules, CA, U.S.A.) was employed for examination. Glyceraldehyde-3-phosphate dehydrogenase (GAPDH) was served as an internal control for relative expression. The primer sequences were synthesized by Shanghai Generay BiotechCo., Ltd. (Shanghai, China). Solubility curve was used to evaluate the reliability of the results of PCR. The 2^−ΔΔ*C*^_t_ was expressed as the ratio of target genes between the control group and the experiment group. The calculation formula was as follows: Δ*C*_t_ = *C*_t__target gene_ − *C*_t__GAPDH_, ΔΔ*C*_t_ = Δ*C*_t__experimental group_ − Δ*C_t_*_control group_ [22]. *C*_t_ referred to the inflection point in the solubility curve. Each experiment was repeated three times to obtain the mean value.

**Table 1:** The primer sequences for RT-qPCR

Gene	Primer sequence (5′–3′)
*Apc*	Forward: CTTCGTGTACGGCAGCTCTT
	Reverse: GCAGTTTCATGCTTGCTCTG
*NF-κB p65*	Forward: GAAGAAGCGACCTGGAG
	Reverse: TCCGGAACACAATGGCCAC
*IκBα*	Forward: CCACGACAGCGGCTTGGACTA
	Reverse: TCCACGATGCCCAGGTAGCC
*MMP-2*	Forward: CTGATAACCTGGATGCAGTCGT
	Reverse: CCAGCCAGTCCGATTTGA
*MMP-9*	Forward: TTCAAGGACGGTCGGTATT
	Reverse: CTCGAGCCTAGACCCAACTTA
*TNF-α*	Forward: TGAGCACAGAAAGCATGATC
	Reverse: CATCTGCTGGTACCACCAGTT
*IL-1β*	Forward: GACCTGTTCTTTGAGGCTGAC
	Reverse: TCCATCTTCTTCTTTGGGTATTGTT
*IL-6*	Forward: GACTGATGTTGTTGAGAGCCACTG
	Reverse: TAGCCACGCCTTCTGTGACTCTAACT
*GAPDH*	Forward: CATCAACGACCCCTTCATTG
	Reverse: GAAGATGGTGATGGGTTTCC

Abbreviation: MMP, matrix metalloproteinase.

### Western blot analysis

IA tissue samples were prepared and treated with 3 ml lysate containing 7 mol/l carbamide, 2 mol/l thiocarbamide, 5 ml/l IPG buffer (pH 3–10), 65 mmol/l dl-DTT, 40 g/l CHAPS, 5 mg/l protease inhibitor, and 10 ml/l trypsin inhibitor. Then the tissues were subjected to phacofragmentation on ice. Next, the samples were centrifuged at 120000 ***g*** for 30 min at 4°C. The obtained supernatant was protein extracts. BCA method was used to determine the protein concentration. The protein was inactivated with 5× serine dehydratase (SDS) lysate (P0013G, Beyotime, Beijing, China) at 100°C for 5 min, followed by electrophoresis on polyacrylamide gel (5% concentration gel and 12% separation gel) with 20 μl sample uploaded. After transfer of membrane, TBS with Tween 20 (TBST) containing 5% BSA was blocked with decolorization table at room temperature for 1 h. Then sealing liquid was aspirated, the membrane was placed into plastic groove with the addition of following primary antibodies: rabbit anti-Apc polyclonal antibody (containing 5% BSA, ab15270, 1:2000), rabbit anti-NF-κB p65 polyclonal antibody (ab19870, 2.5 µg/ml), rabbit anti-IκBα polyclonal antibody (ab7217, 1:2000), rabbit anti-MMP-2 polyclonal antibody (ab97290, 1:2000), rabbit anti-MMP-9 polyclonal antibody (ab138306, 1:500), rabbit anti-TNF-α polyclonal antibody (ab6671, 1:1000), rabbit anti-IL-1β polyclonal antibody (ab9722, 0.2 µg/ml), rabbit anti-IL-6 polyclonal antibody (ab6672, 1:1000), and rabbit anti-p-p65 polyclonal antibody (ab86299, 1:1000) shaken and placed in a freezer at 4°C overnight. All these antibodies were purchased from Abcam Inc. (Cambridge, MA, U.S.A.). On the following day, the membrane was washed with TBST for three times (10 min/time), and added with diluted secondary antibody goat anti-rabbit in the same way (ab6721, Abcam Inc., Cambridge, MA, U.S.A.), incubated for 4–6 h at 4°C, and then the membrane was washed three times with TBST (15 min/time). Electrochemiluminescence of tris (2,2′-bipyridine) ruthenium (II)/tri-n-propylamine (TPA) (Yanhui Biotech Itd., Shanghai, China) were mixed at a ratio of 1:1, dropped off to the nitrocellulose (NC) membrane and developed with. Relative OD value was analyzed for all immunoblotting bands. The experiment was repeated three times to obtain the mean value.

### Statistical analysis

All data were analyzed by SPSS 21.0 (IBM Corp. Armonk, NY, U.S.A.). The measurement data were expressed as mean ± S.D. The equality of variances and homogeneity of variances were analyzed for all the data. Comparisons amongst multiple groups were analyzed by one-way ANOVA. The post-hoc Tukey test was also performed. Enumeration data were presented as numbers or percentages. The correlation of Apc, NF-κB p65, and IκBα p65 aive expression with clinicopathological characteristics of IA was analyzed by Chi-square test. *P*<0.05 was considered to be of statistical significance.

## Results

### Down-regulation of Apc and IκBα and up-regulation of NF-κB p65 are observed in IA

**Figure 1:**
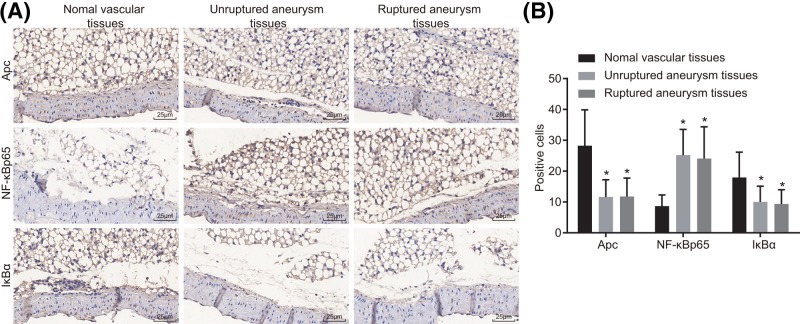
Down-regulation of Apc and IκBα and up-regulation of NF-κB p65 in unruptured and ruptured IA tissues were revealed by immunohistochemstry (× 400) (**A**) Immunohistochemical staining for protein Apc, NF-κB p65, and IκBα in normal vascular tissues, unruptured and ruptured IA tissues. (**B**) Quantitative analysis for positive expression rate; *P<0.05 compared with normal vascular tissues.

The protein expression of Apc, IκBα, and NF-κB p65 was determined by immunohistochemistry. Apc protein was mainly localized in the cytoplasm. The NF-κB p65 and IκBα proteins were essentially localized in the cytoplasm and partially localized in the nucleus. The positive protein expression was mainly light yellow, yellowish-brown, pale brown, and brown (Figure 1). The results showed that compared with normal vessels, positive protein expression of Apc and IκBα was down-regulated while that of NF-κB p65 was up-regulated in unruptured and ruptured IA (all *P*<0.05). The results indicated that reduced expression of Apc and IκBα, and increased expression of NF-κB p65 were identified in unrupture and ruptured IA.

### Positive expression of Apc, NF-κB p65, and IκBα is related with clinicoclinicopathological characteristics of IA

The positive expression of Apc, NF-κB p65, and IκBα with clinicopathological characteristics of IA was examined (Table 2). It was found that protein expression of Apc, NF-κB p65, and IκBα had no correlation to the sex, age, and IA type of patients (all *P*>0.05); whereas they were associated with IA diameter. Amongst them, the positive expression of Apc and IκBα was lower in patients with larger IA diameter, while that of NF-κB p65 was elevated when IA diameter was increased. Namely, when compared with patients whose IA diameter was ≤5 or 5–13 mm, lower positive protein expression of Apc and IκBα and higher positive expression of NF-κB p65 were identified in patients with IA diameter >13 mm (*P*=0.004). It was suggested that decreased expression of Apc and IκBα, and increased NF-κB p65 may link to the diameter of IA.

**Table 2:** The positive expression of Apc, IκBα, and NF-κB p65 protein is involved in clinicopathological characteristics of IA

Characteristics	Case	Apc		*P*-value	IκBα		*P-*value
		Positive	Negative		Positive	Negative	
Age (years)				0.411			0.606
<52	53	25 (47.17%)	10 (18.87%)		14 (26.42%)	39 (73.58%)	
≥52	55	23 (41.82%)	14 (25.45%)		17 (30.91%)	38 (69.09%)	
Sex				0.381			0.881
Male	58	22 (37.93%)	11 (18.97%)		17 (29.31%)	41 (70.69%)	
Female	50	26 (52.00%)	13 (26.00%)		14 (28.00%)	36 (72.00%)	
IA type				0.365			0.819
Unruptured IA	40	17 (42.50%)	7 (17.50%)		12 (30.00%)	28 (70.00%)	
Ruptured IA	68	31 (45.59%)	17 (25.00%)		19 (27.94%)	49 (72.06%)	
IA diameter				0.032			0.044
≤5 mm	44	26 (59.09%)	14 (31.82%)		15 (34.09%)	29 (65.91%)	
5–13 mm	40	18 (45.00%)	9 (22.50%)		14 (35.00%)	26 (65.00%)	
>13 mm	24	4 (16.67%)	1 (4.17%)		2 (8.33%)	22 (91.67%)	

### Up-regulated serum levels of MCP-1, TNF-α, IL-1β, and IL-6 are identified in IA

The levels of MCP-1, TNF-α, IL-1β, and IL-6 in serum were determined by ELISA (Table 3). Compared with the normal group, the levels of MCP-1, TNF-α, IL-1β, and IL-6 significantly increased in both the unruptured IA and ruptured IA groups (*P*<0.05). In contrast with the unruptured IA group, the levels of MCP-1, TNF-α, IL-1β, and IL-6 distinctly increased in the ruptured IA group (*P*<0.05). The results revealed that the up-regulated levels of MCP-1, TNF-α, IL-1β, and IL-6 could promote the formation and rupture of IA.

**Table 3:** ELISA detection indicated that the levels of MCP-1, TNF-α, IL-1β, and IL-6 are increased in serum of patients with IA

Group	Number	MCP-1 (ng/l)	TNF-α (pg/ml)	IL-1β (ng/l)	IL-6 (ng/ml)
Normal	28	134.72 ± 6.59	14.76 ± 2.16	5.83 ± 0.76	57.46 ± 8.94
Unruptured IA	40	162.49 ± 15.89*	39.76 ± 4.32*	37.49 ± 4.59*	81.52 ± 12.38*
Ruptured IA	68	198.62 ± 21.16^*†^	56.17 ± 5.68^*†^	51.48 ± 7.51^*†^	109.23 ± 16.87^*†^

*P<0.05 compared with the normal group. ^†^*P*<0.05 compared with the unruptured IA group.

### siRNA-1 and siRNA-3 are selected for the following experiments

**Figure 2:**
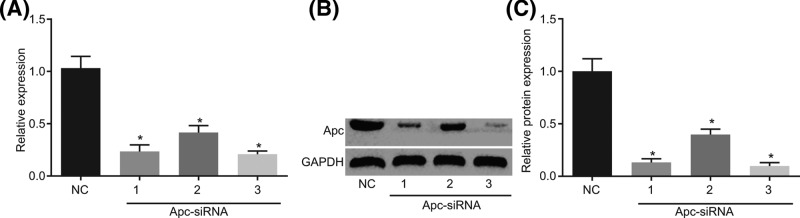
siRNA-1 and siRNA-3 exhibit the highest interference efficiency and are selected for the following experiments (**A**) The mRNA expression of Apc in the siRNA-1, siRNA-2, and siRNA-3 groups detected by RT-qPCR. (**B,C**) Protein expression of Apc in the siRNA-1, siRNA-2, and siRNA-3 groups detected by Western blot analysis; *P<0.05 compared with the NC group.

According to the sequence of Apc, we designed three siRNAs specifically interfering Apc and NC respectively, and detected their interference efficiency by RT-qPCR and Western blot analysis. The results in Figure 2 showed that compared with the NC group, the mRNA and protein expression of Apc in the siRNA-1, siRNA-2, and siRNA-3 groups were significantly decreased (*P*<0.05). Amongst them, siRNA-1 and siRNA-3 had the highest interference efficiency, so we chose the two siRNAs for the following experiments.

### Silencing of Apc increases blood pressure in rats with IA

The blood pressure of rats was determined before operation and 1 week after operation. As shown in Table 4, blood pressure of rats before operation in the blank, NC, Apc-siRNA-1, and Apc-siRNA-3 groups was higher than that in the normal group (*P*<0.05), and the blood pressure was further increased at the 4th and 12th week after operation. The blood pressure of rats had no obvious change during different periods in the normal group. The increased blood pressure of rats in the Apc-siRNA-1 and Apc-siRNA-3 groups all increased at the 1st, 4th, and 12th week after operation in comparison with the blank and NC groups (*P*<0.05). These results indicated that the increased blood pressure in rat model of IA may be associated with silencing of Apc.

**Table 4:** Apc-siRNA contributes to the increase in blood pressure of rat models with IA (mmHg)

Group	*n*	Before operation	1st week after operation	4th week after operation	12th week after operation
Normal	10	99.79 ± 5.89	106.46 ± 10.27	101.38 ± 9.75	109.63 ± 12.34
Blank	10	98.23 ± 9.34	154.52 ± 12.67^*‡^	169.56 ± 20.11^*‡^	174.69 ± 11.84^*‡^
NC	10	101.28 ± 9.89	161.35 ± 13.15^*‡^	175.48 ± 14.34^*‡^	181.54 ± 12.24^*‡^
Apc-siRNA-1	10	97.38 ± 4.89	178.21 ± 11.98^*†‡^	194.57 ± 12.38^*†‡^	197.89 ± 12.43^*†‡^
Apc-siRNA-3	10	100.28 ± 6.97	184.21 ± 8.27^*†‡^	199.57 ± 11.68^*†‡^	203.89 ± 14.53^*†‡^

*P<0.05 compared with the normal group. ^†^*P*<0.05 compared with the blank and NC groups. ^‡^*P*<0.05 compared with the blood pressure pre-surgery.

### Silencing of Apc promotes IA formation and rupture

**Figure 3:**
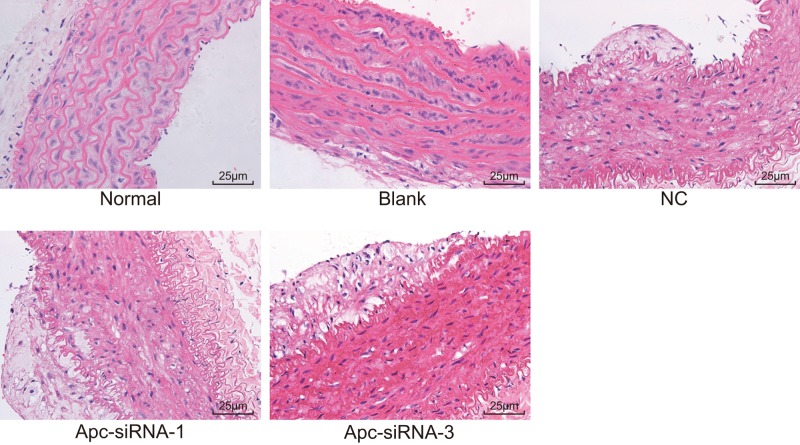
HE staining (×400) demonstrates that silencing of Apc promotes the histopathological changes of IA with slowly disappeared IA endodermis, greatly degenerated VSMC, dramatically declined number and layers of VSMCs, severely fractured elastic fiber, and increased inflammatory cell infiltration

HE staining was conducted for observing the histopathological changes of IA (Figure 3). In normal group, no significant damage of IA endodermis, VSMC, and theca externa was found, and cells in neat arrangement with complete construction. In blank and NC groups, IA endodermis was damaged, VSMC degenerated, the number of VSMC and its layers decreased, with thinner artery wall, fractured elastic fiber, and inflammatory cell infiltration. Compared with the blank and NC groups, IA endodermis disappeared slowly, VSMC greatly degenerated, the number of VSMC and its layers were dramatically declined with thinnest artery wall, severely fractured elastic fiber, and increased inflammatory cell infiltration in the Apc-siRNA-1 and Apc-siRNA-3 groups. Above all, it was indicated that Apc silencing could promote the histopathological changes of IA, suggesting that Apc potentially played a protective role in IA formation and rupture.

### Silencing of Apc promotes the mRNA expression of NF-κB p65, MMP-2, MMP-9, TNF-α, IL-1β, and IL-6, yet inhibits the expression of Apc and IκBα

**Figure 4:**
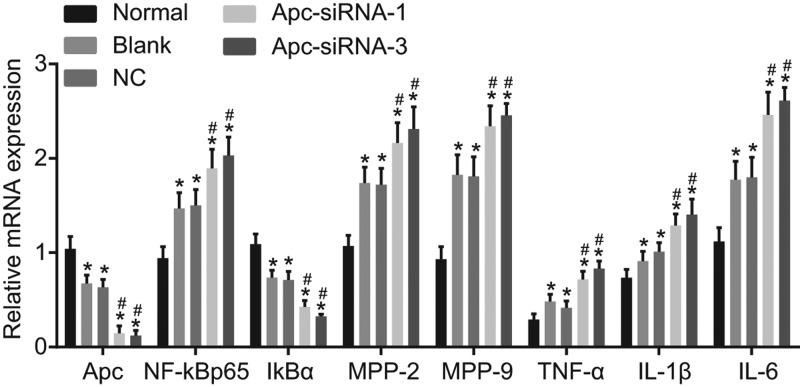
Silencing of Apc contributed to up-regulation of the mRNA expression of NF-κB p65, MMP-2, MMP-9, TNF-α, and IL-1β, and down-regulation of Apc and IκBα according to detection of RT-qPCR *P<0.05 compared with the normal group; ^#^*P*<0.05 compared with the blank and NC groups.

The mRNA expression of NF-κB p65, MMP-2, MMP-9, TNF-α, IL-1β, and IL-6 was determined by RT-qPCR (Figure 4). In comparison with the normal group, mRNA expression of NF-κB p65, MMP-2, MMP-9, TNF-α, IL-1β, and IL-6 was higher in the groups of blank, NC, Apc-siRNA-1, and Apc-siRNA-3, with reduced mRNA expression of Apc and IκBα (all *P*<0.05). When compared with the blank and NC groups, the Apc-siRNA-1 and Apc-siRNA-3 groups showed increased mRNA expression of NF-κB p65, MMP-2, MMP-9, TNF-α, IL-1β, and IL-6 and decreased mRNA expression of Apc and IκBα (all *P*<0.05). So we reached a conclusion that Apc silencing could enhance the expression of NF-κB p65, MMP-2, MMP-9, TNF-α, IL-1β, and IL-6 and inhibit the expression of Apc and IκBα.

### Silencing of Apc enhances the protein expression of NF-κB p65, MMP-2, MMP-9, TNF-α, IL-1β, and IL-6, yet inhibits the expression of Apc and IκBα

**Figure 5:**
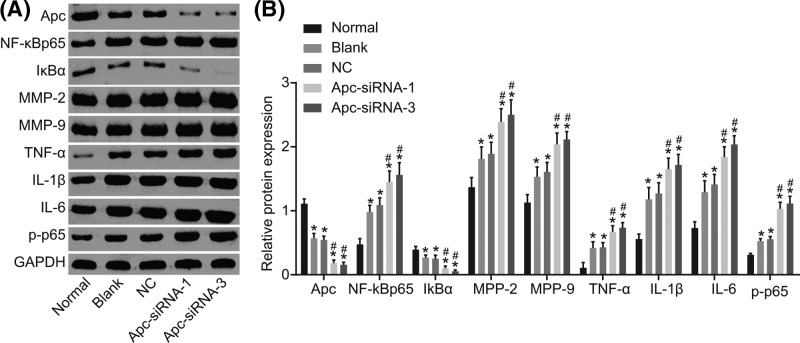
Silencing of Apc contributed to up-regulation of the protein expression of NF-κB p65, MMP-2, MMP-9, TNF-α, and IL-1β, and down-regulation of Apc and IκBα according to detection by Western blot analysis (**A**) Protein bands of NF-κB p65, MMP-2, MMP-9, TNF-α, IL-1β, IL-6, Apc, and IκBα as well as extent of p65 phosphorylation in rats in each group. (**B**) Quantitative analysis of protein expression of NF-κB p65, MMP-2, MMP-9, TNF-α, IL-1β, IL-6, Apc, and IκBα, as well as the extent of p65 phosphorylation of rats in each group; *P<0.05 compared with the normal group; ^#^*P*<0.05 compared with the blank and NC groups.

The protein expression of NF-κB p65, MMP-2, MMP-9, TNF-α, IL-1β, and IL-6 was determined by Western blot analysis. As shown in Figure 5, when compared with normal group, blank, NC, Apc-siRNA-1, and Apc-siRNA-3 groups revealed higher protein expression of NF-κB p65, MMP-2, MMP-9, TNF-α, IL-1β, and IL-6, lower protein expression of Apc and IκBα (all *P*<0.05). In comparison with the blank and NC groups, enhanced protein expression of NF-κB p65, MMP-2, MMP-9, TNF-α, IL-1β, and IL-6, as well as extent of p65 phosphorylation and declined protein expression of Apc and IκBα were found in the Apc-siRNA-1 and Apc-siRNA-3 groups (all *P*<0.05). Therefore, we concluded that silencing of Apc could elevate the protein expression of NF-κB p65, MMP-2, MMP-9, TNF-α, IL-1β, and IL-6, as well as extent of p65 phosphorylation and decrease Apc and IκBα, thus promoting IA formation and rupture.

### Silencing of Apc up-regulates the levels of MCP-1, TNF-α, IL-1β, and IL-6 in brain tissue homogenate

The levels of MCP-1, TNF-α, IL-1β, and IL-6 in brain tissue homogenate were evaluated by ELISA. As shown in Table 5, compared with the normal group, levels of MCP-1, TNF-α, IL-1β, and IL-6 were increased in rat brain tissue homogenate in the groups of blank, NC, Apc-siRNA-1, and Apc-siRNA-3 (all *P*<0.05). When compared with the blank and NC groups, the levels of MCP-1, TNF-α, IL-1β, and IL-6 in the Apc-siRNA-1 and Apc-siRNA-3 groups were significantly enhanced (all *P*<0.05). Therefore, Apc silencing could increase levels of MCP-1, TNF-α, IL-1β, and IL-6 and contribute to IA formation and rupture.

**Table 5:** ELISA detection demonstrates that Apc-siRNA increases levels of MCP-1, TNF-α, IL-1β, and IL-6 in brain tissue homogenate of rat models with IA

Group	*n*	MCP-1 (pg/ml)	TNF-α (ng/ml)	IL-1β (ng/l)	IL-6 (pg/ml)
Normal	10	54.28 ± 9.23	1.27 ± 0.36	69.38 ± 7.21	91.68 ± 16.28
Blank	10	78.49 ± 10.23*	2.54 ± 0.49*	89.26 ± 12.47*	128.67 ± 19.08*
NC	10	87.76 ± 11.34*	2.58 ± 0.58*	96.48 ± 13.42*	142.51 ± 20.48*
Apc-siRNA-1	10	109.27 ± 16.59^*†^	4.31 ± 1.62^*†^	118.53 ± 17.84^*†^	182.94 ± 23.92^*†^
Apc-siRNA-3	10	112.27 ± 15.54^*†^	4.67 ± 1.24^*†^	120.11 ± 12.49^*†^	186.94 ± 27.33^*†^

*P<0.05 compared with the normal group. ^#^All *P*<0.05 compared with the blank and NC groups.

## Discussion

IAs and IA rupture are one of the causes of brain injury and mortality [23]. Importantly, the major risk factors of IA development are highly associated with the proinflammatory immune responses [24]. Interestingly, aberrant methylation of the *Apc* gene is associated with the inflammatory responses [25]. In this study, we identified the expression of Apc and activation of NF-κB signaling pathway in IA and further explored the underlying effects of them on the formation and rupture of IA.

First of all, we revealed that the protein expression of Apc was reduced and the NF-κB signaling pathway was activated in the unruptured and ruptured IAs. Apc defines T-cell differentiation and anti-inflammatory function via microtubule-mediated nuclear factor of activated T-cell localization [26]. Apc serves a key function in maintaining the polarized radial glial scaffold in the development of brain and it is significant for the construction of cerebral cortex in mammals [27]. In addition, a study has suggested the mutation in exon 15 of the Apc gene in a case of brain metastasis [28]. Notably, we found that lower protein expression of Apc and activated NF-κB signaling pathway were correlated with larger sizes of IAs. Aneurysm walls are commonly characterized by activated inflammatory responses, and NF-κB has been reported to be the main transcription factor mediating the expression of inflammation-related genes in the IAs [29]. Through the inhibition of the MAPKs/NF-κB signaling pathway mediated peripheral and cerebral inflammatory response, paeoniflorin, a neuroprotective treatment for stroke, plays a protective role in ischemia injury [30]. Also, the inhibition of the activation of NF-κB prevents the initiation and progression of lung and breast cancers [31]. Activation of transcription factor NF-κB is frequently encountered in tumor cells and contributes to aggressive tumor growth and resistance to chemotherapy and ionizing radiation during cancer treatment [32].

The loss-of-function of *Apc* gene is associated with nuclear accumulation of β-catenin and promotes the constitutive activation of Wnt signaling [14]. This study provided evidence that silencing of Apc induced by siRNA promoted the activation of the NF-κB signaling pathway in rat models of IA. Wnt/β-catenin signaling is pivotal for the development and regeneration of tissues, whereas NF-κB is a critical mediator of inflammation. A previous investigation shows that the activation of NF-κB by CARD4 may be a component of the innate immune response [33]. Both Wnt and NF-κB signaling pathways are boosted in colon adenocarcinoma tissues of Apc Olfm4 double-mutant mice [34]. Recent evidence suggests that these two signaling pathways cross-modulate each other’s activities and functions [35]. A previous study argues that Apc, β-catenin, and NF-κB are functionally interrelated in the carcinogenesis of gastric cancer [36]. More importantly, persistent epithelial NF-κB activation facilitates the functional loss of Apc in intestinal epithelial cells through up-regulation of iNOS [37].

Moreover, the serum levels of MCP-1, TNF-α, IL-1β, and IL-6 were elevated in rat models of IAs. Multiple studies have indicated that inflammation remarkably contributes to aneurysm formation and rupture [6,7]. The activation of NF-κB has been demonstrated to be one of the initiating risk factors leading to the formation and development of IA. MCP-1 has also been reported to induce macrophage infiltration and adhesion in the artery wall of IA, and resulted in the formation and development of IA [38]. The enlargement and rupture of IA was promoted by Ets-1, a mediator for vascular inflammation and remodeling, by inducing the levels of MCP-1 in VSMCs [39]. Reducing TNF-α action in the IA wall carries a beneficial effect on attenuating aneurysm progression by inhibiting inflammation as well as arterial remodeling [40]. Inhibition of IL-1β through genetic and pharmacological strategies decreased formation and enlargement of thoracic aortic aneurysms, suggesting that IL-1β may be a potential target for thoracic aortic aneurysm treatment [41]. Nishihara et al. [42] stated that the IL-6 activity is closely implicated in continuous cellular infiltration in abdominal aortic aneurysm and possibly induces human abdominal aortic aneurysm. Corroborating to this, our study suggested that silencing of Apc induced by siRNA facilitated the formation and rupture of IAs in rat models through up-regulating blood pressure of rats and the levels of MCP-1, TNF-α, IL-1β, and IL-6 in rat brain tissue homogenate.

The key findings of the present study provide evidence emphasizing that poor expression of *Apc* gene and activation of the NF-κB signaling pathway were involved in the formation and rupture of IAs in rat models. The *Apc* gene was revealed to inhibit the activation of the NF-κB signaling pathway, by which mechanism the formation and rupture of IAs were attenuated through reducing inflammatory responses induced by activation of the NF-κB signaling pathway. A greater understanding of the pathogenesis of IA would provide a fundamental basis for the development of new therapies to prevent the rupture in the first place or modify its progression. The specific mechanism of how silencing of Apc affects NF-κB activation warrants further study.

## Abbreviations

Apc: adenomatous polyposis coli
CARD4: Caspase recruitment domain protein 4
DDW: double distilled water
DON: Deoxynivalenol
DSA: digital subtraction angiography
GAPDH: glyceraldehyde-3-phosphate dehydrogenase
HE: Hematoxylin–Eosin
HRP: horseradish peroxidase
IA: intracranial aneurysm
IL: interleukin
iNOS: inducible nitric oxide synthase
MAPK: mitogen-activated protein in kinase
MCP-1: monocyte chemoattractant protein-1
NC: negative control
NF-κB: nuclear factor-κB
OD: optical density
RT-qPCR: reverse transcription quantitative PCR
SP: streptavidin-perosidase
TBST: TBS with Tween 20
TNF-α: tumor necrosis factor-α
VSMC: vascular smooth muscle cell

## References

[1] Caranci F., Briganti F., Cirillo L., Leonardi M. and Muto M. (2013) Epidemiology and genetics of intracranial aneurysms. Eur. J. Radiol. 82, 1598–1605 10.1016/j.ejrad.2012.12.026 23399038

[2] Cebral J.R., Mut F., Weir J. and Putman C.M. (2011) Association of hemodynamic characteristics and cerebral aneurysm rupture. AJNR Am. J. Neuroradiol. 32, 264–270 10.3174/ajnr.A2274 21051508 PMC3070915

[3] Loewenstein J.E., Gayle S.C., Duffis E.J., Prestigiacomo C.J. and Gandhi C.D. (2012) The natural history and treatment options for unruptured intracranial aneurysms. Int. J. Vasc. Med. 2012, 898052 22500236 10.1155/2012/898052 PMC3303690

[4] Johnston S.C., Dowd C.F., Higashida R.T., Lawton M.T., Duckwiler G.R., Gress D.R. (2008) Predictors of rehemorrhage after treatment of ruptured intracranial aneurysms: the Cerebral Aneurysm Rerupture After Treatment (CARAT) study. Stroke 39, 120–125 10.1161/STROKEAHA.107.495747 18048860

[5] Benaissa A., Barbe C. and Pierot L. (2015) Analysis of recanalization after endovascular treatment of intracranial aneurysm (ARETA trial): presentation of a prospective multicenter study. J. Neuroradiol. 42, 80–85 10.1016/j.neurad.2014.04.003 25012816

[6] Chalouhi N., Ali M.S., Jabbour P.M., Tjoumakaris S.I., Gonzalez L.F., Rosenwasser R.H. (2012) Biology of intracranial aneurysms: role of inflammation. J. Cereb. Blood Flow Metab. 32, 1659–1676 10.1038/jcbfm.2012.84 22781330 PMC3434628

[7] Sawyer D.M., Pace L.A., Pascale C.L., Kutchin A.C., O’Neill B.E., Starke R.M. (2016) Lymphocytes influence intracranial aneurysm formation and rupture: role of extracellular matrix remodeling and phenotypic modulation of vascular smooth muscle cells. J. Neuroinflammation 13, 185 10.1186/s12974-016-0654-z 27416931 PMC4946206

[8] Aoki T., Fukuda M., Nishimura M., Nozaki K. and Narumiya S. (2014) Critical role of TNF-alpha-TNFR1 signaling in intracranial aneurysm formation. Acta Neuropathol. Commun. 2, 34 10.1186/2051-5960-2-34 24685329 PMC3974421

[9] Kurki M.I., Hakkinen S.K., Frosen J., Tulamo R., von und zu Fraunberg M., Wong G. (2011) Upregulated signaling pathways in ruptured human saccular intracranial aneurysm wall: an emerging regulative role of Toll-like receptor signaling and nuclear factor-kappaB, hypoxia-inducible factor-1A, and ETS transcription factors. Neurosurgery 68, 1667–1675, 10.1227/NEU.0b013e318210f001 21336216

[10] Chang Y.S., Lin C.Y., Yang S.F., Ho C.M. and Chang J.G. (2016) Analysing the mutational status of adenomatous polyposis coli (APC) gene in breast cancer. Cancer Cell Int. 16, 23 10.1186/s12935-016-0297-2 27028212 PMC4810512

[11] Fodde R., Smits R. and Clevers H. (2001) APC, signal transduction and genetic instability in colorectal cancer. Nat. Rev. Cancer 1, 55–67 10.1038/35094067 11900252

[12] Li Y., Lauriola M., Kim D., Francesconi M., D’Uva G., Shibata D. (2016) Adenomatous polyposis coli (APC) regulates miR17-92 cluster through beta-catenin pathway in colorectal cancer. Oncogene 35, 4558–4568 10.1038/onc.2015.522 26804172 PMC4960006

[13] Pecina-Slaus N., Nikuseva Martic T., Zeljko M. and Bulat S. (2011) Brain metastases exhibit gross deletions of the APC gene. Brain Tumor Pathol. 28, 223–228 10.1007/s10014-011-0030-8 21442240

[14] Wang X.P., O’Connell D.J., Lund J.J., Saadi I., Kuraguchi M., Turbe-Doan A. (2009) Apc inhibition of Wnt signaling regulates supernumerary tooth formation during embryogenesis and throughout adulthood. Development 136, 1939–1949 10.1242/dev.033803 19429790 PMC2680115

[15] Huang G.L., Luo Q., Rui G., Zhang W., Zhang Q.Y., Chen Q.X. (2013) Oncogenic activity of retinoic acid receptor gamma is exhibited through activation of the Akt/NF-kappaB and Wnt/beta-catenin pathways in cholangiocarcinoma. Mol. Cell. Biol. 33, 3416–3425 10.1128/MCB.00384-13 23798555 PMC3753848

[16] Wong E.T. and Tergaonkar V. (2009) Roles of NF-kappaB in health and disease: mechanisms and therapeutic potential. Clin. Sci. (Lond.) 116, 451–465 10.1042/CS20080502 19200055

[17] Catrysse L. and van Loo G. (2017) Inflammation and the metabolic syndrome: the tissue-specific functions of nf-kappab. Trends Cell Biol. 27, 417–429 10.1016/j.tcb.2017.01.006 28237661

[18] Cheng W.T. and Wang N. (2013) Correlation between MMP-2 and NF-kappa B expression of intracranial aneurysm. Asian Pac. J. Trop. Med. 6, 570–573 10.1016/S1995-7645(13)60098-X 23768831

[19] Aoki T., Kataoka H., Nishimura M., Ishibashi R., Morishita R. and Miyamoto S. (2012) Regression of intracranial aneurysms by simultaneous inhibition of nuclear factor-kappaB and Ets with chimeric decoy oligodeoxynucleotide treatment. Neurosurgery 70, 1534–1543, 10.1227/NEU.0b013e318246a390 22186838

[20] Yamamoto R., Aoki T., Koseki H., Fukuda M., Hirose J., Tsuji K. (2017) A sphingosine-1-phosphate receptor type 1 agonist, ASP4058, suppresses intracranial aneurysm through promoting endothelial integrity and blocking macrophage transmigration. Br. J. Pharmacol. 174, 2085–2101 10.1111/bph.13820 28409823 PMC5466536

[21] Makino H., Tada Y., Wada K., Liang E.I., Chang M., Mobashery S. (2012) Pharmacological stabilization of intracranial aneurysms in mice: a feasibility study. Stroke 43, 2450–2456 10.1161/STROKEAHA.112.659821 22798328 PMC3429647

[22] Tuo Y.L., Li X.M. and Luo J. (2015) Long noncoding RNA UCA1 modulates breast cancer cell growth and apoptosis through decreasing tumor suppressive miR-143. Eur. Rev. Med. Pharmacol. Sci. 19, 3403–3411 26439035

[23] Tulu S., Mulino M., Pinggera D., Luger M., Wurtinger P., Grams A. (2015) Remote ischemic preconditioning in the prevention of ischemic brain damage during intracranial aneurysm treatment (RIPAT): study protocol for a randomized controlled trial. Trials 16, 594 10.1186/s13063-015-1102-6 26714784 PMC4696326

[24] Zhang H.F., Zhao M.G., Liang G.B., Yu C.Y., He W., Li Z.Q. (2016) Dysregulation of CD4(+) T cell subsets in intracranial aneurysm. DNA Cell Biol. 35, 96–103 10.1089/dna.2015.3105 26667180

[25] Van der Auwera I., Van Laere S.J., Van den Bosch S.M., Van den Eynden G.G., Trinh B.X., van Dam P.A. (2008) Aberrant methylation of the Adenomatous Polyposis Coli (APC) gene promoter is associated with the inflammatory breast cancer phenotype. Br. J. Cancer 99, 1735–1742 10.1038/sj.bjc.6604705 18841156 PMC2584952

[26] Aguera-Gonzalez S., Burton O.T., Vazquez-Chavez E., Cuche C., Herit F., Bouchet J. (2017) Adenomatous polyposis coli defines treg differentiation and anti-inflammatory function through microtubule-mediated NFAT localization. Cell Rep. 21, 181–194 10.1016/j.celrep.2017.09.020 28978472

[27] Yokota Y., Kim W.Y., Chen Y., Wang X., Stanco A., Komuro Y. (2009) The adenomatous polyposis coli protein is an essential regulator of radial glial polarity and construction of the cerebral cortex. Neuron 61, 42–56 10.1016/j.neuron.2008.10.053 19146812 PMC2804250

[28] Pecina-Slaus N., Majic Z., Musani V., Zeljko M. and Cupic H. (2010) Report on mutation in exon 15 of the APC gene in a case of brain metastasis. J. Neurooncol. 97, 143–148 10.1007/s11060-009-0001-7 19711014

[29] Pawlowska E., Szczepanska J., Wisniewski K., Tokarz P., Jaskolski D.J. and Blasiak J. (2018) NF-kappaB-mediated inflammation in the pathogenesis of intracranial aneurysm and subarachnoid hemorrhage. Does autophagy play a role? Int. J. Mol. Sci. 19, pii: E1245, 10.3390/ijms19041245 PMC5979412 29671828

[30] Guo R.B., Wang G.F., Zhao A.P., Gu J., Sun X.L. and Hu G. (2012) Paeoniflorin protects against ischemia-induced brain damages in rats via inhibiting MAPKs/NF-kappaB-mediated inflammatory responses. PLoS ONE 7, e49701 10.1371/journal.pone.0049701 23166749 PMC3498223

[31] Dey A., Wong E., Kua N., Teo H.L., Tergaonkar V. and Lane D. (2008) Hexamethylene bisacetamide (hmba) simultaneously targets akt and mapk pathway and represses nf kappab activity: Implications for cancer therapy. Cell Cycle 7, 3759–3767 10.4161/cc.7.23.7213 19029824

[32] Li F. and Sethi G. (2010) Targeting transcription factor nf-kappab to overcome chemoresistance and radioresistance in cancer therapy. Biochim. Biophys. Acta 1805, 167–180 20079806 10.1016/j.bbcan.2010.01.002

[33] Bertin J., Nir W.J., Fischer C.M. (1999) Human card4 protein is a novel ced-4/apaf-1 cell death family member that activates nf-kappab. J. Biol. Chem. 274, 12955–12958 10.1074/jbc.274.19.12955 10224040

[34] Liu W., Li H., Hong S.H., Piszczek G.P., Chen W. and Rodgers G.P. (2016) Olfactomedin 4 deletion induces colon adenocarcinoma in apc(min/+) mice. Oncogene 35, 5237–5247 10.1038/onc.2016.58 26973250 PMC5057043

[35] Ma B. and Hottiger M.O. (2016) Crosstalk between Wnt/beta-Catenin and NF-kappaB signaling pathway during Inflammation. Front. Immunol. 7, 378 10.3389/fimmu.2016.00378 27713747 PMC5031610

[36] Kim B., Byun S.J., Kim Y.A., Kim J.E., Lee B.L., Kim W.H. (2010) Cell cycle regulators, APC/beta-catenin, NF-kappaB and Epstein-Barr virus in gastric carcinomas. Pathology 42, 58–65 10.3109/00313020903356392 20025482

[37] Shaked H., Hofseth L.J., Chumanevich A., Chumanevich A.A., Wang J., Wang Y. (2012) Chronic epithelial NF-kappaB activation accelerates APC loss and intestinal tumor initiation through iNOS up-regulation. Proc. Natl. Acad. Sci. U.S.A. 109, 14007–14012 10.1073/pnas.1211509109 22893683 PMC3435160

[38] Liu Y., Zhang Y., Dai D. and Xu Z. (2014) Expression of NF-kappaB, MCP-1 and MMP-9 in a cerebral aneurysm rabbit model. Can. J. Neurol. Sci. 41, 200–205 10.1017/S0317167100016589 24534031

[39] Aoki T., Kataoka H., Nishimura M., Ishibashi R., Morishita R. and Miyamoto S. (2010) Ets-1 promotes the progression of cerebral aneurysm by inducing the expression of MCP-1 in vascular smooth muscle cells. Gene Ther. 17, 1117–1123 10.1038/gt.2010.60 20428211

[40] Jayaraman T., Paget A., Shin Y.S., Li X., Mayer J., Chaudhry H. (2008) TNF-alpha-mediated inflammation in cerebral aneurysms: a potential link to growth and rupture. Vasc. Health Risk Manag. 4, 805–817 10.2147/VHRM.S2700 19065997 PMC2597764

[41] Johnston W.F., Salmon M., Pope N.H., Meher A., Su G., Stone M.L. (2014) Inhibition of interleukin-1beta decreases aneurysm formation and progression in a novel model of thoracic aortic aneurysms. Circulation 130, S51–S59 10.1161/CIRCULATIONAHA.113.006800 25200056 PMC5097450

[42] Nishihara M., Aoki H., Ohno S., Furusho A., Hirakata S., Nishida N. (2017) The role of IL-6 in pathogenesis of abdominal aortic aneurysm in mice. PLoS ONE 12, e0185923 10.1371/journal.pone.0185923 28982132 PMC5628902

