# Expression of Concern: Apc gene suppresses intracranial aneurysm formation and rupture through inhibiting the NF-κB signaling pathway mediated inflammatory response

**DOI:** 10.1042/BSR-2018-1909_EOC

**Published:** 2024-10-04

**Keywords:** Apc gene, Inflammatory response, intracranial aneurysm, NF-κB signaling pathway

The Editorial Office has been made aware of potential issues surrounding the scientific validity of this paper “Apc gene suppresses intracranial aneurysm formation and rupture through inhibiting the NF-κB signaling pathway mediated inflammatory response” DOI: 10.1042/BSR20181909, hence has issued an expression of concern to notify readers whilst the Editorial Office investigates. It has been noted that Figure 3 Normal panel and parts of Figure 3 NC panel seem to appear in a subsequent paper by Gao et al, 2020 (DOI: 10.1186/s11671-020-03357-2) [retracted].

Additionally, the Editorial Office has concerns that during the revision process, the labels for two panels in Figure 3 changed condition. The authors were contacted regarding the concerns, however so far the Editorial Office has not received a response.

